# The environmental yeast *Cryptococcus liquefaciens* produces capsular and secreted polysaccharides with similar pathogenic properties to those of *C. neoformans*

**DOI:** 10.1038/srep46768

**Published:** 2017-04-25

**Authors:** Glauber R. de S. Araújo, Gustavo J. C. Freitas, Fernanda L. Fonseca, Paulo Emilio C. Leite, Gustavo Miranda Rocha, Wanderley de Souza, Daniel A. Santos, Susana Frases

**Affiliations:** 1 Laboratório de Ultraestrutura Celular Hertha Meyer, Instituto de Biofísica Carlos Chagas Filho, Federal University of Rio de Janeiro, Rio de Janeiro, Brazil; 2 Departamento de Microbiologia, Instituto de Ciências Biológicas, Universidade Federal de Minas Gerais, Minas Gerais, Brazil; 3 Centro de Desenvolvimento Tecnológico em Saúde. Fundação Oswaldo Cruz. Rio de Janeiro, Brazil

## Abstract

Invasive fungal infections, including cryptococcosis, are a growing threat to immunocompromised patients. Although *Cryptococcus neoformans* and *Cryptococcus gattii* are the main agents of human cryptococcosis, opportunistic infections by environmental species, such as *C. liquefaciens*, have been observed recently. The main *Cryptococcus* virulence factor is the production and secretion of polysaccharides (PS). Previously, we showed that both species produce PS of similar composition. Here, we examined the ultrastructure and biological activity of capsular and secreted PS from *C. liquefaciens*, and yeast pathogenicity to an invertebrate host, in comparison with *C. neoformans*. Ultrastructural analysis by high-resolution microscopy showed that both species produce large and complex capsules. PS from both species had indistinguishable effects on phagocytosis levels, NO production and the secretion of a variety of immune mediators. Challenge with *C. liquefaciens* or *C. neoformans* led to complete lethality of *G. mellonella* larvae. Treatment with *C. liquefaciens* PS could not protect mice against infection with *C. neoformans*. We conclude that polysaccharides of the environmental yeast *C. liquefaciens* have strikingly similar ultrastructural and biological properties to those of *C. neoformans*, highlighting the importance of monitoring the emergence of new fungal pathogens for which thermotolerance may be an important transitional step towards pathogenesis in humans.

The rates of human invasive fungal infections have increased significantly (by 200%) in the last three decades^1^, particularly among intensive care patients and immunocompromised individuals, such as those with the acquired immunodeficiency syndrome (AIDS)^2^,^3^,^4^,^5^,^6^. *Cryptococcus neoformans* is one of the main fungal species that cause invasive disease in humans. Together, *C. neoformans* and the related species *C. gattii* are responsible for ~650,000 human deaths each year, worldwide^7^.

The most important and best characterized virulence factor of *C. neoformans* is the ability to produce polysaccharides (PS), which form an external capsule anchored to the cell surface, and are also secreted^8^,^9^,^10^. Monoclonal antibodies against the PS capsule are protective in an animal model of cryptococcosis^11^, and molecules mimicking structural aspects of the cryptococcal PS are potential candidates for a vaccine against cryptococcosis^12^, highlighting the fundamental role of the PS in infection.

An ‘accidental’ role of the PS capsule is to protect *C. neoformans* against phagocytosis by immune system cells^11^. The capsule also promotes virulence by triggering immune system cell apoptosis, inhibiting different aspects of the host immune response (including antibody production, leukocyte migration, complement activity and antigen presentation) and protecting yeast cells against reactive oxygen species^10^,^13^,^14^,^15^,^16^,^17^,^18^. On the other hand, secreted PS also modulates host immune responses, potentiating infection^19^. Cryptococcal PS activates Toll-like receptors, leading to the production of pro-inflammatory cytokines, including TNF-α and IL-12, and increasing the antimicrobial potential and antigen presentation by host phagocytes^20^.

Although the biological function of the capsule is well described, less is known about the physical and structural characteristics of the capsular PS^10^,^21^,^22^,^23^. Obtaining precise structural information on the capsule in its native state is challenging, since the hydrophilic capsular PS is invisible by light microscopy (due to its low refractive index in aqueous medium) and is also susceptible to dehydration with solvents^24^, a required step in most high-resolution microscopy techniques. Thus, the capsule is easily ruptured or denatured during preparation for conventional electron microscopy^25^,^26^,^27^,^28^, which makes it difficult to obtain valuable information on the structure of the capsular PS, at a macromolecular level.

Extensive analysis by a variety of physico-chemical methods and optical tweezers, combined with the use of advanced electron microscopy techniques, provided key insights into capsular PS ultrastructure and other properties^23^. These data show that cryptococcal capsular and secreted PS are formed of molecules with different physico-chemical and ultrastructural properties, and that PS molecules are highly complex and branched polymeric structures whose orientation varies in different regions of the capsule^22^. Furthermore, Cordero and co-workers (2011) provided strong evidence that the degree of PS branching and conformation affects the biological activity of the *C. neoformans* capsular PS^21^.

In addition to *C. neoformans* and *C. gattii*, which are important human pathogens, approximately 35 other *Cryptococcus* species have been described^11^. Although non-*neoformans* and non-*gattii* species have traditionally been considered environmental yeasts that are not pathogenic to humans, a significant increase in the incidence of opportunistic human infections by these species has been observed in recent years^29^,^30^. Infections caused by these species are presumably acquired from the environment, through a variety of potential sources that serve as reservoirs, including bird excrement, trees, food (cheese and fruit), soil and water^11^. While the increased incidence of non-*neoformans* and non-*gattii* human infections may stem from improved laboratory detection and the higher incidence of immunocompromised individuals, it is also possible that global warming progressively increases thermal tolerance among species of environmental fungi, leading to adaptation to the warmer conditions found in the human host^31^,^32^.

Most studies on the capsule have been conducted in *C. neoformans*, due to the presumably low pathogenic potential of other species of the genus. Recently, the first cases of human fungemia by *C. liquefaciens* in immunocompromised patients have been described^33^,^34^. The authors described an extremely rare case of polymicrobial meningitis in a patient with HIV caused by *C. liquefaciens* and *Mycobacterium tuberculosis*. They alerted that it is difficult to distinguish between *C. liquefaciens* and related species on a structural basis, in the lack of sequence data. For this reason, they suggested that some reported cases of disseminated cryptococcosis may have been incorrectly identified, which indicates that not only *C. liquefaciens* but also other related species could be more common than currently thought. More recently, *C. liquefaciens* was also reported in a case of central venous catheter-related fungemia in a patient without HIV infection who recovered after receiving fluconazole and voriconazole. In this case, the *C. liquefaciens* strain was resistance to 5-fluorocytosine^33^,^34^.

Interestingly, we showed in a previous study that the PS of the environmental yeast *C. liquefaciens* has similar composition to that of *C. neoformans*. However, physico-chemical analysis of the *C. liquefaciens* polysaccharides in comparison with those of *C. neoformans* revealed that capsular structures in pathogenic *Cryptococcus* species and environmental species share similar features, but also display significant rheological differences that could influence their potential virulence^35^. Thus, it is important to examine further the PS molecules (as the main virulence factors) and the infectivity of *C. liquefaciens*, and this analysis may provide useful insights on human pathogenicity development among other environmental *Cryptococcus* species.

In this work, an important objective was to evaluate the ‘hidden’ potential of environmental strains to become pathogenic; thus, the choice of a non-pathogenic strain here had the advantage of highlighting traits that might facilitate pathogenicity, once thermotolerance is acquired. For this, we compared the ultrastructure of the *C. liquefaciens* capsule PS with that of *C. neoformans*, using advanced microscopy techniques that allow the capsule to be observed in a state nearer-to-native^28^. We also compared the biological activities of *C. liquefaciens* and *C. neoformans* secreted PS towards mammalian macrophages, examining phagocytosis and NO production, and analyzing the secretion of a panel of immune mediators by the human THP-1 macrophage lineage, upon exposure to secreted PS. In addition, we used an invertebrate infection model to compare the infectivity of the two *Cryptococcus* species.

## Results

### Both capsular and secreted PS from *C. neoformans* and *C. liquefaciens* consist of ultrastructurally similar fibers

To compare the size and ultrastructure of the capsules of *C. neoformans* and *C. liquefaciens*, yeasts from these species were cultured under the same growth conditions and analyzed by light microscopy, and also by advanced scanning electron microscopy techniques – namely high-resolution scanning electron microscopy (HRSEM) and helium ion microscopy (HIM). These techniques can achieve high resolution - in the nanometer scale - and allow sensitive samples to be visualized in a closer-to-native state, particularly during HIM imaging, which is performed without conductive coating.

**Figure 1:**
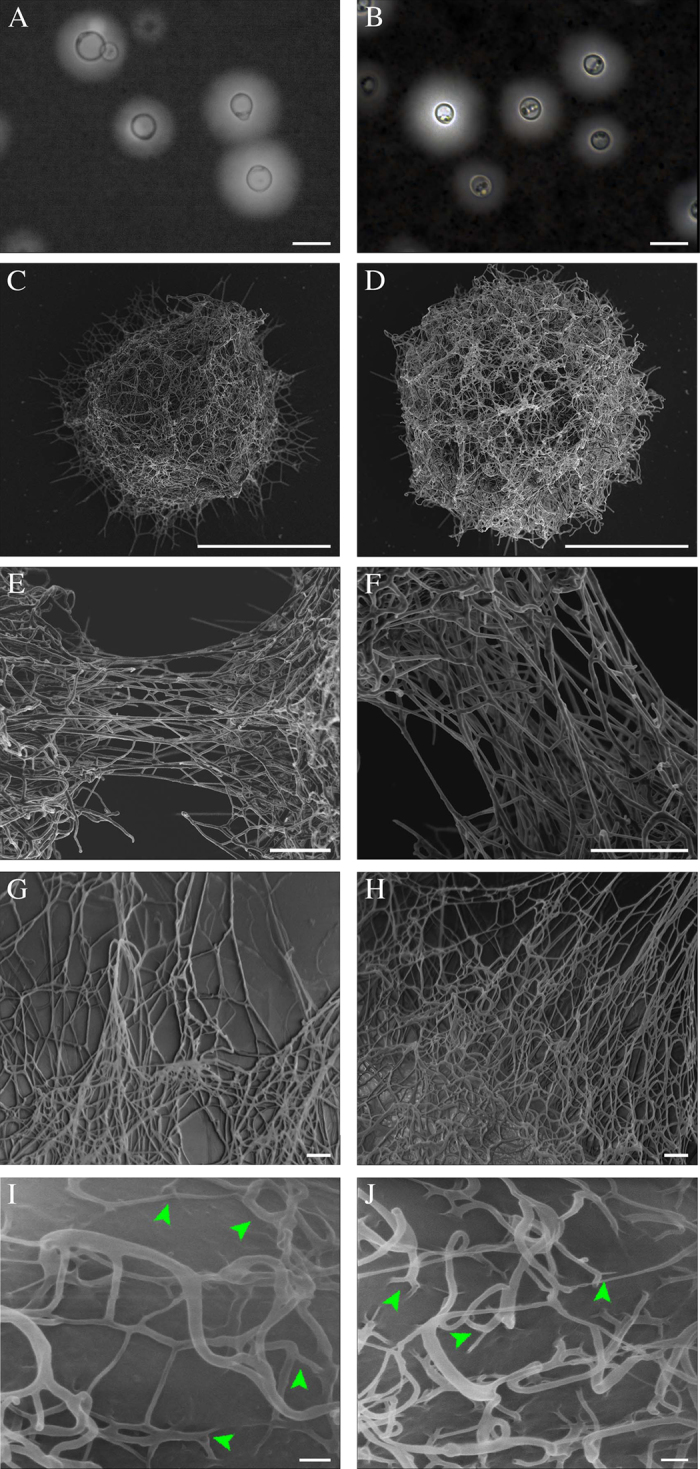
Strutural characterization of *Cryptococcus neoformans* and *C. liquefaciens* capsule. (**A**,**B**) Light microscopy of *C. neoformans* (**A**) and *C. liquefaciens* (**B**) cells after negative staining with India ink, showing the capsule as a light halo of approximately 5.1 ± 2.2 and 4.5 ± 2.8 μm, in *C. neoformans* and *C. liquefaciens*, respectively (p = 0.094, N = 100 cells). (**C**–**H**) High resolution scanning electron microscopy (HRSEM) of carbon-coated PS capsule formed by *C. neoformans* (**C**,**E** and **G**) and *C. liquefaciens* (**D**,**F** and **H**). (**I**,**J**) Helium ion microscopy (HIM) of the surface of *C. neoformans* (**I**) and *C. liquefaciens* (**J**) cells (with no metal coating). Arrowheads indicate triskelion structures. Scale bars: 10 μm (**A** and **B**); 5 μm (**C** and **D**); 500 nm (**E** and **F**); 200 nm (**G** and **H**); and 100 nm (**I** and **J**).

As measured from light microscopy images of cells negatively stained with India ink (Fig. 1A,B), capsule thickness did not vary significantly between the two strains (5.1 ± 2.2 and 4.5 ± 2.8 μm, in *C. neoformans* and *C. liquefaciens*, respectively; p = 0.094).

When observed by HRSEM/HIM, both *C. neoformans, C. liquefaciens* yeast cells exhibited branched capsules (Fig. 1C,H). For both species, HRSEM/HIM imaging showed branched PS fibers with different lengths and widths interacting with each other to form a heterogeneous asymmetrical *microgel*-*like* network of high structural complexity (Fig. 1E–J). The fibers forming this network could be placed in the following categories according to their diameter: 12.6 ± 3.1 nm; 20.4 ± 2.4 nm; 34.6 ± 1.7 nm; 42.5 ± 4.6 nm; 60.5 ± 5.3 nm. Based on fiber length, width and heterogeneity, as seen by HRSEM, we could not identify clear structural differences in the microgel organization of the capsule, between *C. liquefaciens* and *C. neoformans* (Fig. 1E–H).

When visualized by HIM - which allows high-resolution observation of cell surfaces, while avoiding artefacts from metal coating^28^ - capsules from both *C. liquefaciens* and *C. neoformans* had identical PS fiber heterogeneity, with progressive increments in fiber diameter as a consequence of the lateral interaction between multiple fibers. Measurements of fiber widths from HIM images showed a distribution of fibers in categories of 5.23 ± 1.51 nm, 12.49 ± 1.42 nm, 25.55 ± 2.25 nm, 31.17 ± 1.62 nm and >310.4 nm. We could not detect differences between the categories of fiber widths observed in the different species (Fig. 1I,J). We also observed ‘triskelion’ structures appearing to anchor PS fibers to the surface of the fungal cell wall, in *C. liquefaciens*, as described for *C. neoformans*^35^ (Fig. 1I,J arrowhead).

**Figure 2:**
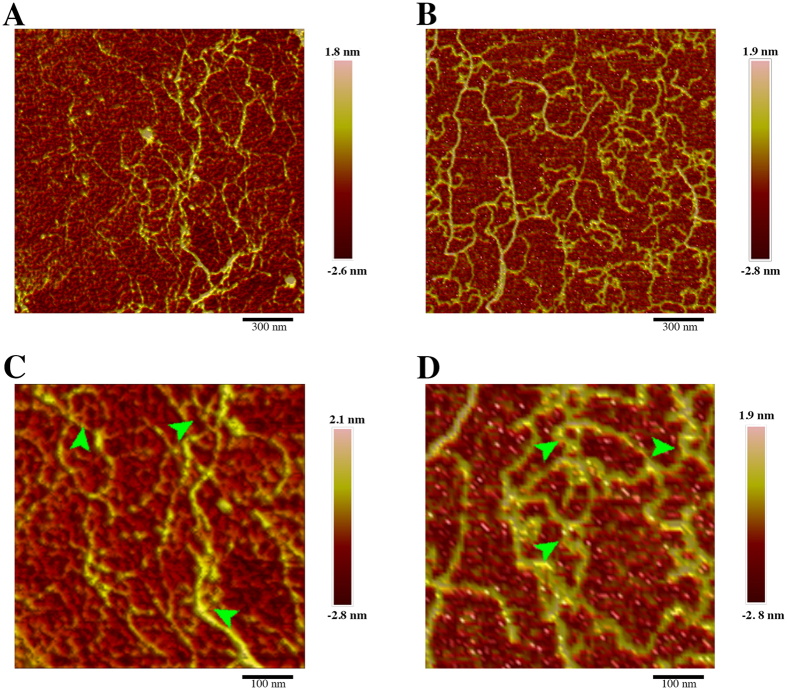
Atomic force microscopy of *Cryptococcus* secreted polysaccharides (secreted-PS). Topographical images obtained at PeakForce tapping mode (in air) of *C. neoformans* (**A** and **C**) and *C. liquefaciens* (**B** and **D**) secreted-PS. Arrowheads indicate handle-shaped structures branching off polysaccharide fibers. Scale bars: 300 nm (**A**,**B**) and 100 nm (**C**,**D**).

We examined the organization of secreted-PS molecules from both species using atomic force microscopy (AFM), which allowed us to obtain topographic images in the nanometer scale without critical point drying. AFM images of secreted-PS showed linear molecules with average length of 675 ± 14 nm, for *C. neoformans*, and 704 ± 44 nm, for *C. liquefaciens* (Fig. 2). Branch ‘handles’ – globular structures at one end of each fiber (arrowheads in Fig. 2), similar to those previously described in branched commercial purified PS visualized by AFM^36^ - were more common in *C. neoformans* PS, but were also present in *C. liquefaciens* PS (Fig. 2).

### Secreted PS from *C. liquefaciens* and *C. neoformans* trigger similar levels of macrophage phagocytosis and NO production

**Figure 3:**
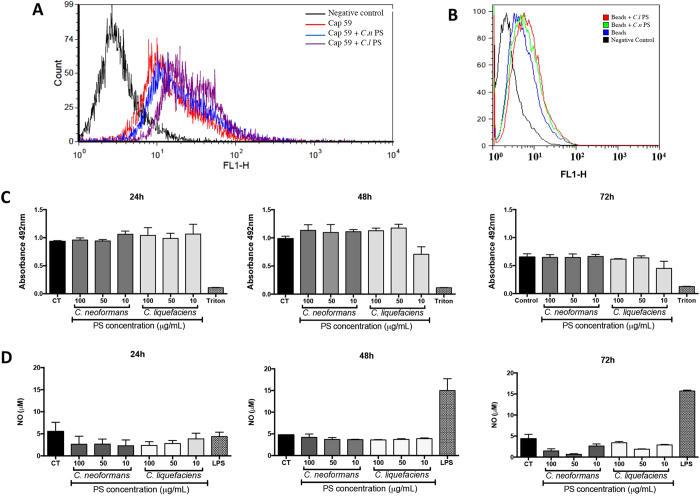
Phagocytosis by murine macrophages of acapsular *Cryptococcus neoformans* cells (cap59 mutant) or beads coated with secreted PS. (**A**,**B**) Macrophages were allowed to interact, for 72 h, with FITC-labelled Cap59 (**A**) or polystyrene beads (**B**) (coated with secreted-PS from *C. neoformans (Cn*-PS) or *C. liquefaciens (Cl*-PS), and then analyzed by flow cytometry. Non-infected macrophages were used as controls (CT). (**C**) Cell viability (by the XTT assay) and nitric oxide production (**D**) of murine macrophages exposed to secreted-PS from *C. neoformans* and *C. liquefaciens* for 24, 48 and 72 h. Treatment with Triton X-100 and LPS were used as positive controls for cell viability loss and macrophage activation, respectively. In (**C** and **D**) data represent mean ± SD of 3 independent experiments.

Since one of the functions of the *C. neoformans* PS is to protect yeasts against internalization by phagocytes, we analyzed the effect of *C. liquefaciens* PS in the interaction with mammalian host cells *in vitro*, and compared this effect with that of the *C. neoformans* PS. To study the effect of the PS independently from that of other components produced by yeast cells, we used acapsular *C. neoformans* mutant cells (Cap59 mutant) and polystyrene beads coated with secreted-PS, instead of capsular yeast cells. After 72 h of interaction of coated or uncoated beads (which were also FITC-labelled) and murine macrophages, flow cytometry analysis showed very similar profiles for phagocyte populations that interacted with particles coated with *C. liquefaciens* or *C. neoformans* PS (Fig. 3A and B). We also analyzed the effects of internalization of PS-coated cells and beads on macrophage viability (by the XTT assay) and on the production of the key antimicrobial effector nitric oxide (NO). After 72 h of interaction, we did not observe statistically significant reductions in the viability of macrophages that interacted with secreted-PS from *C. liquefaciens* or *C. neoformans*, on coated beads or Cap59 cells (Fig. 3C). Similarly, internalization of particles coated with secreted-PS from *C. neoformans* and *C. liquefaciens* stimulated similar levels of NO production by macrophages to those observed in the negative control (non-infected macrophages), after 24, 48 and 72 hours of interaction (Fig. 3D).

### Evaluation of secreted mediators induced by *C. neoformans* and *C. liquefaciens* secreted-PS in THP-1 human macrophages

**Figure 4:**
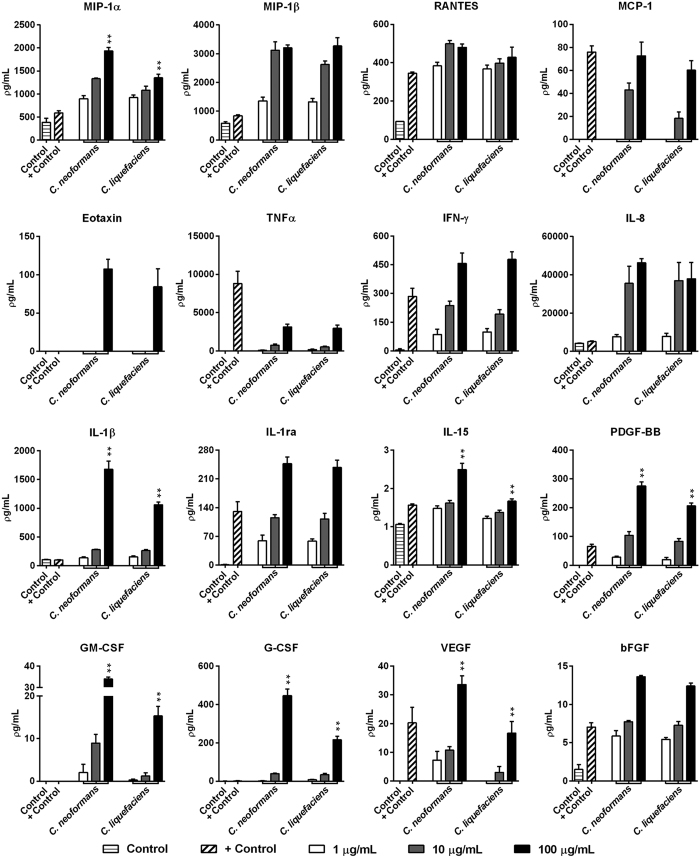
Production of secreted mediators by TPH-1 human macrophages incubated for 24 h with 1, 10 or 100 μg/ml of secreted polysaccharides (secreted-PS) from *C. neoformans* or *C. liquefaciens*. Secreted mediator levels in culture supernatants were determined using the Magpix xMAP multiplex system (Biorad Laboratories Inc., Hercules, CA, USA). Control, untreated cells; + Control, cells incubated with 20 ng/mL TNFα. Data represent mean ± SEM of 4 independent experiments. **p < 0.01 relative to cells challenged by 100 μg/ml *C. neoformans* vs. *C. liquefaciens* (Student’s *t*-test).

To investigate the secretion pattern of different immune system modulators, human macrophages were treated with different concentrations of secreted-PS (1, 10 and 100 μg/mL) from both species. Analysis of a panel of secreted mediators was performed using a multiplex magnetic bead system (Fig. 4). This panel included 27 mediators such as chemokines, cytokines and growth factors. Treatment with PS from *C. neoformans* or *C. liquefaciens* stimulated the production of 16 of the 27 analytes. The levels of the following mediators increased in a dose-dependent manner after stimulation by *C. liquefaciens* and *C. neoformans* PS: the chemokines MIP-1α, MIP-1β, RANTES, MCP-1 and Eotaxin, the cytokines TNFα, IFN-γ, IL-8, IL-1β, IL-1ra and IL-15, and the growth factors PDGF-BB, GM-CSF, G-CSF, VEGF and bFGF (Fig. 4). Overall, PS molecules from both species triggered a potent inflammatory response in human macrophages (Fig. 4). Indeed, 100 μg/mL *C. neoformans* PS led to higher levels of MIP-1α, IL-1β, IL-15, PDGF-BB, GM-CSF, G-CSF and VEGF compared with 100 μg/mL *C. liquefaciens* PS (**p < 0.01). These results demonstrate that *C. neoformans* PS molecules display a stronger pro-inflammatory potential.

### Cells and secreted PS from *C. neoformans* and *C. liquefaciens* have identical effects on invertebrate host survival

**Figure 5:**
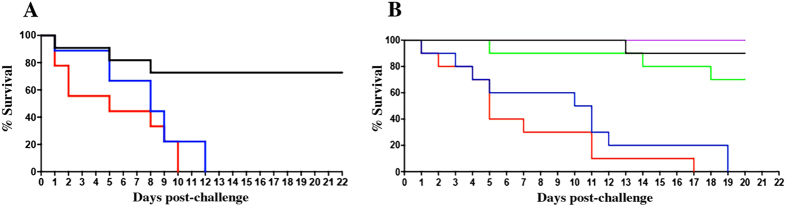
Survival of *Galleria mellonella* larvae after challenge with *C. neoformans* (blue) or *C. liquefaciens* (red) whole cells (**A**) or secreted PS (coated beads (**B**). There were no significant differences between groups infected with cells (p-value = 0.2446) or treated with PS from different *Cryptococcus* species (p-value = 0.2446). n = 20 larvae/group, in two independent experiments. Black- untreated larvae; purple – PBS-treated animals; green - uncoated beads.

To examine possible differences in virulence between *C. liquefaciens* and *C. neoformans*, both yeast species were used to infect larvae of the moth *G. mellonella*. This model has been used for studying virulence and the action of antifungal drugs against *C. neoformans*^37^. While 70% of larvae remained alive up to 15 days after PBS inoculation (negative control), challenge with either *C. liquefaciens* or *C. neoformans* led to death of all larvae by day 12 post-infection, with no significant differences in survival rates between infected groups (Fig. 5A). Similarly, challenge with polystyrene beads coated with secreted PS from *C. liquefaciens* or *C. neoformans* led to the death of 100% of larvae by day 17 post-infection (compared with 75 and 85% survival at the same stage, with uncoated beads or PBS, respectively). Statistical analysis showed no significant differences between the effects of PS from the two species (Fig. 5B).

### Analysis of the immunoprotective potential of *C. liquefaciens* secreted-PS against infection by *C. neoformans*

**Figure 6:**
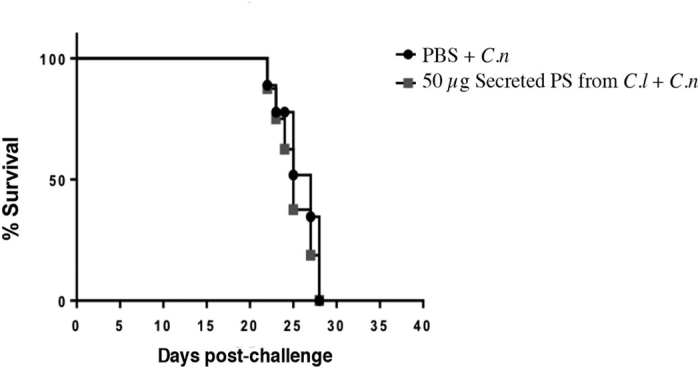
Effect of secreted PS from *C. liquefaciens* on mouse survival following *C. neoformans* infection. C57/BL6 mice were administered PBS (black circles) or secreted-PS from *C. liquefaciens* (gray squares), by intraperitoneal injection, 15 and 7 days pre-infection and 1, 7 and 15 days post-infection with *C. neoformans (C.n*.).

Given the strong similarities in secreted PS from *C. liquefaciens* and *C. neoformans* – in both ultrastructure and the potential to elicit biological responses (in mammalian cells and in an invertebrate host) - we tested whether secreted PS from *C. liquefaciens* could provide protection against infection by *C. neoformans*. C57/BL6 mice were repeatedly sensitized with *C liquefaciens* PS (or with PBS, as a negative control), both before and after a challenge with *C. neoformans* cells, and mouse survival was examined daily. Infection with *C. neoformans* led to the death of all animals by day 26 post-infection, in the group treated with *C. liquefaciens* PS and in the PBS-treated group (Fig. 6). Statistical analysis showed no significant differences between the sensitized and non-sensitized groups, showing that *C. liquefaciens* secreted PS could not elicit a protective immune response against infection by *C. neoformans*.

## Discussion

Capsule PS is the first barrier to the interaction between *Cryptococcus* spp. and the surrounding environment, and represents the main virulence factor of these yeast pathogens^11^. Although we previously demonstrated that the polysaccharide capsules of *C. neoformans* and *C. liquefaciens* have similar chemical composition and different physical characteristics, *C. liquefaciens* pathogenicity to humans was not previously considered since the fungus is not normally able to grow at the temperatures encountered in the human host^35^. In this study we showed that both the capsules and the secreted polysaccharides from these two *Cryptococcus* species look visually indistinguishable using high resolution microscopy. Physiological tests with the *C. liquefaciens* strain revealed a lack of urea hydrolysis and an inability to generate pigmentation after incubation with L-dopa for 20 days, suggesting that this species does not produce melanin (Figure S1). We also observed a growth deficiency of *C. liquefaciens* at temperatures above 35 °C (Figure S1). However, recent clinical reports have identified *C. liquefaciens* as a pathogen in immunocompromised patients^33^,^34^, highlighting the importance of studying the pathogenic potential of environmental *Cryptococcus* species.

Given that the lack of virulence of many species of the *Cryptococcus* genus can be explained (at least in part) by the lack of mammalian thermotolerance or/and the immune status of the host, it is uncertain whether capsular production and release by these organisms are comparable to those of the pathogenic cryptococci. Therefore, we examined whether *C. liquefaciens*, despite being an environmental strain, was pathogenic to mammalian host cells. In particular, we focused on the role of the polysaccharide capsule, analyzing the ultrastructure and biological activity of both the capsular and the secreted PS of *C. liquefaciens*, using the pathogenic species *C. neoformans* as a comparison.

For many years in the *Cryptococcus* field, the branched structures observed in images of Cryptococcus capsular PS were considered artefacts of dehydration and collapse of adjacent PS molecules^11^. Recently our group demonstrated that the capsular PS from *C. neoformans* is in fact branched, by using advanced microscopy techniques that allow the PS to be observed in a nearer-to-native state^21^,^28^. Here, observations of capsular and secreted PS using these techniques showed clear ultrastructural similarities in the branched arrangement of capsular PS molecules from *C. liquefaciens* and *C. neoformans*, providing strong evidence that the capsules from both species are assembled as branched structures, despite their homogeneous appearance when viewed by light microscopy (Fig. 1). HRSEM, HIM and AFM images show branched polysaccharide fibers of different lengths (from 100 to 1500 nm) and widths (from 10 to 95 nm), which interact to form a heterogeneous microgel-like network of high structural complexity. These results are in agreement with our previous data showing that capsular PS molecules from both *C. liquefaciens* and *C. neoformans* have a very low ‘shape factor’ (i.e., a large difference between the radius of gyration and the hydrodynamic radius), which is characteristic of molecules with a hyperbranched higher order structure^21^,^35^,^38^. We also observed stratification in images of the capsule, with a ‘dense core’ (virtually impermeable to India ink, in light microscopy preparations) and an outer core in the distal edge of the capsule (Fig. 1). The capsule ultrastructure and conformation observed here are associated with the ability of the capsular PSs to self-aggregate, which is believed to be an important factor for capsule formation^10^,^35^,^39^.

Most of the observations of capsular architecture have been based on samples coated with metals (such as Au and Pt, commonly used for SEM imaging). Our HIM data showing *C. liquefaciens* and *C. neoformans* cells without conductive coating demonstrate that PS branching is not an artifact of the coating process^28^. As expected, carbon-coated PS fibers, as observed by HRSEM, appeared thicker than the non-coated PS fibers seen by HIM (Fig. 1). Moreover, HIM imaging of *C. liquefaciens* and *C. neoformans* also allowed us to resolve, on the surface of the fungal cell wall, ‘triskelion’ structures that represent anchoring of cross-linked PS molecules to cell wall glucans^28^,^40^,^41^.

Previously, we demonstrated that there are significant physico-chemical differences between secreted and capsular PS in *C. neoformans*^42^, showing that these molecules have distinct properties and should, therefore, be analyzed independently. AFM images of secreted PS from *C. liquefaciens* showed linear fibers containing a handle-like structure at the end (Fig. 2), similar to that described for *C. neoformans*^28^,^43^. For both species, although PS fibers were linear, as expected for these sugars, fibers interacted to form the aggregated, branched structures that were observed in images of both secreted and capsular PS.

As observed for other natural polysaccharides - where conformation affects immunomodulatory and antitumor effects^44^,^45^,^46^,^47^,^48^ - the structure and conformation of cryptococcal PS molecules are associated with their biological activity^49^,^50^,^51^. However, the increased susceptibility of *C. liquefaciens* to internalization and killing by amebae (an important environmental predator) compared with *C. neoformans*^35^ shows that ultrastructural similarities in secreted and capsule PS between these species may not be predictive of comparable biological properties.

Nevertheless, we show here that the biological effects of the capsular and secreted PS of *C. liquefaciens* are highly similar to those of the *C. neoformans* PS, in assays of macrophage interaction and secretion stimulation, and during infection in an invertebrate host model. When interacting with mammalian macrophages, the *C. neoformans* PS capsule acts as both a chemical and a physical barrier for recognition by macrophages^52^; however, when internalized, yeast cells are capable of surviving and replicating inside phagocytes^10^,^13^,^14^,^15^,^16^,^17^,^18^. On the other hand, *C. liquefaciens* was internalized with similar efficiency compared to *C. neoformans*, and the environmental yeast suppressed the NO-mediated fungicidal activity of macrophages (Fig. 3), as described for *C. neoformans*. Structure similarities between the PS capsules of *C. liquefaciens* and *C. neoformans* could influence the ability of murine macrophage to internalize and stimulate NO production and survival, after interaction with the PS from these fungi.

Exposure of human macrophages to *C. liquefaciens* and *C. neoformans* PS led to the production of 16 out of the 27 secreted mediators tested here, including a dose-dependent increase in the secretion of key mediators of Th1-type responses. Such alteration on inflammatory pathways may upregulate the established inflammatory response, during infection. In complex systems, it would affect cell homeostasis and possibly increase susceptibility to cell death.

In an assay of virulence against an invertebrate host – represented by larvae of the moth *G. mellonella* – infection with *C. liquefaciens* or *C. neoformans* led to identical survival rates, with 100% death of infected animals by day 10–12 (p-value = 0.27; Fig. 5A), showing that these cryptococcal species have comparable potential for generating systemic lethal infections in an animal model. A similar effect in survival was observed after treatment with beads coated in *C. liquefaciens* or *C. neoformans* PS (p-value = 0.24 Fig. 5B), which indicates that PS molecules contribute substantially to the lethality of whole cells towards an invertebrate host. Thus, the similarity in the capsular ultrastructure and composition - described here and in a previous study from our group^35^ - are associated with identical biological response elicited by the PS of *C. liquefaciens* and *C. neoformans*.

*C. neoformans* is the most common invasive fungal pathogen in the HIV-infected population^7^; however, no vaccines have been approved for use against cryptococcosis. Despite the similarities in structure, composition and biological responses between capsular and secreted PS molecules of *C. liquefaciens* and *C. neoformans*, the secreted PS from *C. liquefaciens* could not protect mice against cryptococcosis caused by *C. neoformans* (Fig. 6), with no significant differences between the sensitized and non-sensitized groups. These data suggest that key antigenic differences exist between the PS molecules from these two species.

## Conclusion

We conclude that the striking similarities in composition and structure between (capsule and secreted) PS molecules of *C. liquefaciens* and *C. neoformans* are matched by comparable behavior in several key biological activities, including the interaction with mammalian phagocytes, the stimulation of NO and immune modulator production by macrophages, and the survival in an animal model. As an initial study this work suggests remarkable similarities in the capsule ultrastructure and virulence, between single isolates of *C. neoformans* and *C. liquefaciens*. Further studies with more strains and approaches are necessary to understand the complex dynamics of assembly and remodelling of the capsule of *Cryptococcus* sp. Our work provides evidence that fungal virulence factors are essential for yeast survival in the environment, because fungi traditionally regarded as non-pathogenic to humans have virulence factors similar to those of their pathogenic relatives. These non-pathogenic fungi could emerge as new causative agents of disease upon the acquisition of thermotolerance to mammalian temperatures, which may be facilitated by the selective pressure of global warming. Also, emerging pathogens might be not be sensitive to the available antifungals, potentiating the threat of novel fungal diseases. The initial data presented here on a comparison of two strains may contribute to guide the analyzes of new strains or species, to produce a wider picture of the relationship between capsule structure, composition and dynamics and the interaction of *Cryptococcus* spp with the host.

## Methods

### Cryptococcus strains

The following *Cryptococcus* spp. strains were used in this work: *C. neoformans var. grubii* H99 (ATCC 208821, clinical isolate, kindly donated by Arturo Casadevall, Johns Hopkins Bloomberg School of Public Health, Baltimore, Maryland, USA); a *C. liquefaciens* strain isolated from the snail *Achatina fulica*^35^; and the acapsular *C. neoformans var. grubii* mutant Cap59^53^; kindly donated by Arturo Casadevall, Johns Hopkins Bloomberg School of Public Health, Baltimore, Maryland, USA). The strains were maintained in glycerol stocks at −80 °C and grown on rich Sabouraud media at 30 °C.

### Induction of capsule production

Capsule production was induced by growing yeast cells in minimal medium containing 15 mM glucose, 10 mM MgSO_4_7H_2_O, 29 mM KH_2_PO_4_, 13 mM glycine, and 3 μM thiamine, at 30 °C, for 5 days.

### Purification of secreted polysaccharides

Secreted capsule polysaccharides were purified by ultrafiltration using an Amicon system with a cutoff of 100 kDa (Millipore, Danvers, MA), as described previously^39^. The concentration of polysaccharides in filtered solutions was determined by the phenol-sulfuric method^54^, using glucose as a standard.

### Coating of yeasts and beads with secreted polysaccharides

Cap59 cells (in exponential phase of growth) and polystyrene beads of 3 ± 0.15 μm (CV 5%; Polysciences, Inc. Warminster, PA, USA) were incubated for 12 hours with 10 μg/ml of secreted-PS from *C. neoformans* H99 or *C. liquefaciens* in PBS, at room temperature and under constant agitation. Uncoated beads were used as a negative control. Then, sample were washed in PBS three times to remove non-adhered PS. To confirm binding, samples were visualized by conventional scanning electron microscopy as described by Araujo *et al*.^28^.

### Light microscopy

To measure capsule thickness, cells were centrifuged at 6708 × g for 5 min, negatively stained with India ink and imaged in an AXIO Lab.A1 light microscope (ZEISS, Germany). The capsule thickness (i.e., the distance between the cell wall and the outer limit of the capsule) was measured from random images of at least 100 cells, using the ImageJ software 1.40 g (NIH, Bethesda, MD, USA).

### High-resolution scanning electron microscopy (HRSEM)

For HRSEM imaging, yeast cells were washed three times in PBS and fixed in 2.5% glutaraldehyde type I, in 0.1 M sodium cacodylate buffer (pH 7.2), for 1 h at room temperature. After fixation, cells were washed in 0.1 M sodium cacodylate buffer (pH 7.2) containing 0.2 M of sucrose and 2 mM of MgCl_2_. Then, cells were adhered to coverslips coated with 0.01% poly-L-lysine (Sigma-Aldrich, St. Louis, Mi, USA), for 20 min at room temperature, dehydrated in ethanol (30, 50 and 70%, for 5 min, then 95% and 100% twice, for 10 min), and subjected to critical point drying in an MS DPC 300 (Leica, Wetzlar, Germany). Then, samples were coated with carbon and analyzed using a Magellan high-resolution electron microscope (FEI, Hillsboro, USA), operating at 1 kV. Widths of 100 fibers from different positions in each samples were measured, in individual images from three independent replicates, using the ImageJ software (NIH, Bethesda, MD, USA).

### Helium ion microscopy (HIM)

Cells were fixed, dehydrated and critical point dried as described above (see “High-resolution scanning electron microscopy”), and left uncoated. Samples were imaged in a Zeiss Orion NanoFab Helium Ion Microscope (Carl Zeiss, Peabody, MA, USA), and secondary electron images of 2048 × 2048 pixels were acquired at 35 kV.

### Atomic Force Microscopy (AFM)

To observe secreted polysaccharides by AFM, 20 μL aliquots of 10 μg/ml solutions of secreted-PS (in PBS) were placed directly in mica (0.21 mm of thickness and 10 mm of diameter), dried in an atmosphere with high concentration of nitrogen gas, and immediately observed in a Dimension FastScan atomic force microscope (Bruker, Santa Barbara, USA). Images were acquired in PeakForce Tapping mode, in air, with a 90° reading angle and a 2 Hz scan rate matrix of 512 × 521 pixels. Image analysis was performed using the Nanoscope Analysis Software (Bruker, Santa Barbara, USA).

### Phagocytosis assays

For phagocytosis assays, murine macrophages (ATCC RAW 264.7) were cultured at 37 °C (with 5% CO_2_), in 24-well plates (10^5^ cells/well) containing Dulbecco’s modified Eagle medium (DMEM, Sigma-Aldrich, St. Louis, Mi, USA) supplemented with 10% inactivated fetal calf serum). FITC-labelled yeasts/beads coated with exogenous polysaccharide as described above (see “*Coating of yeasts and beads with secreted polysaccharides*”) were incubated with macrophages at a ratio of 10 yeast/beads per host cell, for 1 to 18 h, at 37 °C. To remove non-adherent yeast/beads, plates were washed several times with sterile PBS. Infected macrophages were removed by scraping and then analyzed in a FACSCalibur flow cytometer (BD Biosciences 298, San Jose, CA). Data were analyzed using CellQuest (BD Biosciences, San Jose, CA) and WinMDI (Salk Flow Cytometry, La Jolla, CA, USA). Non-infected macrophages were used as controls (CT). Data were subjected to statistical analysis by the Student’s t-test (two-tailed).

### Nitric Oxide (NO) determination

The murine macrophage cell line ATCC RAW 264.7 was cultivated in complete Dulbecco’s Modified Eagle Medium (DMEM) with 10% heat-inactivated (56 °C for 30 min) fetal calf serum (Gemini Bio-products, Woodland, CA, USA), 10% NCTC-109 medium (Gibco), and 1% MEM non-essential amino acids (Gibco-Invitrogen 11360), at 37 °C in a 10% CO_2_ atmosphere. Secreted PSs isolated from *C. neoformans* and *C. liquefaciens* (at final concentrations of 100, 50 and 10 μg/mL) were added to wells of 24-well plates (1 mL total volume/well) containing 1 × 10^6^ macrophages/well, and incubated for 16 h at 37 °C, in a 10% CO_2_ atmosphere. After incubation, supernatants were collected and subjected to a quick centrifugation step, to remove cell debris. NO production was analyzed in supernatants using the Griess Reagent System (Promega, Madison, WI). Macrophage-like cells stimulated with 0.5 μg/mL lipopolysaccharide (LPS) or in the absence of PS were used as controls. NO production was calculated as the difference in NO_2_ concentration relative to the difference in PS amount (in nmoles). Experiments were performed in triplicates. Non-infected macrophages were used as controls (CT).

### XTT assay

For macrophage viability studies the XTT colorimetric method was performed after interaction of macrophages with PS-containing solutions. Readings were performed in a spectrophotometer at λ = 492 nm. PBS was used as a “blank”, and non-infected macrophages were used as controls (CT).

### Survival assays in *Galleria mellonella*

Larvae of the moth *G. mellonella* were selected according to size (1.8 to 2.0 cm) and the absence of any pigmentation marks, for reproducible results. An average of 20 animals per experimental group were inoculated with 10 μL of suspensions containing 10^6^ *C. liquefaciens* or *C. neoformans* cells in PBS, or with polystyrene beads coated with secreted PS (as described above; see *Coating of yeasts and beads with secreted polysaccharides*). Prior to injection, the paw area was cleaned with 70% ethanol. After injection, larvae were placed in 90-mm glass plates and incubated at 25 °C. The numbers of dead larvae were recorded daily. Untreated larvae (not manipulated) and larvae inoculated with PBS only were used as controls. Kaplan-Meier survival curves were produced using Graph Pad Prism 5 (La Jolla, CA, USA). Data are representative of two independent experiments.

### Protection assay in mice

Male C57/BL6 mice were sensitized intraperitoneally with 50 μL of secreted-PS (1 mg/ml) from *C. liquefaciens*, both before (15 and 7 days) and after (1, 7 and 15 days) a challenge with 1 × 10^4^ *C. neoformans* cells via intratracheal inoculation. PBS was used as a negative control. The protocol for animal studies was approved by the Ethics Committee for Animal Experimentation from the Federal University of Minas Gerais (Comissão de Ética no Uso de Animais - CETEA/UFMG, Brazil; Protocol 204/2015) and animal experiments were performed in strict agreement with the Brazilian Federal Law 11,794, which establishes procedures for the scientific use of animals. All mice were housed in clean bedding (five mice per cage) with food and water *ad libitum*, in a controlled environment with a 12 h light/dark cycle, at 23 °C. All mice were monitored twice daily. For intratracheal inoculation, mice were anesthetized by intraperitoneal injection of ketamine hydrochloride (80 mg Kg^−1^) and xylazine (100 mg Kg^−1^) in sterile saline. Any mice that appeared moribund (e.g. presenting intense piloerection, convulsions, and/or lack of locomotor activity) were euthanized immediately (under anaesthesia) by cervical dislocation performed by experienced animal handlers.

### Multiplex assay for secreted mediators

THP-1 monocytic cells (TIB-202 from ATCC, USA; mycoplasma-free, using MycoAlert Mycoplasma Detection Kit – Lonza, Bazel, Switzerland, and at passage numbers ≤ 20) were seeded at 5 × 10^3^ cells/well in 96-well plates, and maintained as subconfluent monolayers in complete RPMI medium (with 10% FBS). Cells were differentiated into macrophages by incubation in medium containing 0.5 μM phorbol 12-myristate 13-acetate (PMA, Sigma-Aldrich, St. Louis, Mi, USA) for 24 h at 37 °C, and 5% CO_2_. After differentiation, cell supernatants were discarded, and cells were incubated for 24 h in complete medium containing 1, 10 or 100 μg/mL of *C. neoformans* or *C. liquefaciens* secreted PS. Control cells did not receive any type of treatment (Control). As a positive control for the release of pro-inflammatory mediators, cells were incubated with 20 ng/mL TNFα (+Control).

After exposure to secreted PS or TNFα, the levels of secreted mediators in culture supernatants were determined using the Luminex xMAP system, comprising a 27-Plex panel magnetic bead kit (for IL-1β, IL-1ra, IL-2, IL-4, IL-5, IL-6, IL-7, IL-8, IL-9, IL-10, IL-12 (p70), IL-13, IL-15, IL-17, eotaxin, bFGF, GCSF, GM-CSF, IFN-γ, IP-10, MCP-1 (MCAF), MIP-1α, MIP-1β, PDGF-BB, RANTES, TNFα, VEGF), as previously described^55^. The concentration of each secreted product was estimated using the xPONENT software version 4.2 (Biorad Laboratories Inc., Hercules, CA, USA).

### Statistical Analysis

Data were subjected to statistical analysis by Student’s t-tests using the Graph Pad Prism 5 (La Jolla, CA, USA), with p < 0.05 considered statistically significant.

## Additional Information

**How to cite this article:** Araújo, G. R. S. *et al*. The environmental yeast *Cryptococcus liquefaciens* produces capsular and secreted polysaccharides with similar pathogenic properties to those of *C. neoformans. Sci. Rep.* **7**, 46768; doi: 10.1038/srep46768 (2017).

**Publisher's note:** Springer Nature remains neutral with regard to jurisdictional claims in published maps and institutional affiliations.

## Supplementary Material

Supplementary Figures
