# Supplementary material for: The environmental yeast Cryptococcus liquefaciens produces capsular and secreted polysaccharides with similar pathogenic properties to those of C. neoformans
Source: Sci Rep. 2017 Apr 25;7:46768. doi: 10.1038/srep46768 (PMC5404263; doi:10.1038/srep46768)
Supplement: Supplementary Figures [file srep46768-s1.pdf]

The environmental yeast *Cryptococcus liquefaciens* produces capsular and secreted polysaccharides with similar pathogenic properties to those of *C. neoformans*.

Glauber R de S. Araújo[1], Gustavo J.C. Freitas[2], Fernanda L. Fonseca[3], Paulo Emilio C Leite[1], Gustavo Miranda Rocha[1], Wanderley de Souza[1], Daniel A. Santos[2], Susana Frases[1*]

[1]Laboratório de Ultraestrutura Celular Hertha Meyer, Instituto de Biofísica Carlos Chagas Filho, Federal University of Rio de Janeiro, Rio de Janeiro, Brazil.
[2] Departamento de Microbiologia, Instituto de Ciências Biológicas, Universidade Federal de Minas Gerais, Minas Gerais, Brazil.
[3] Centro de Desenvolvimento Tecnológico em Saúde. Fundação Oswaldo Cruz. Rio de Janeiro, Brazil.

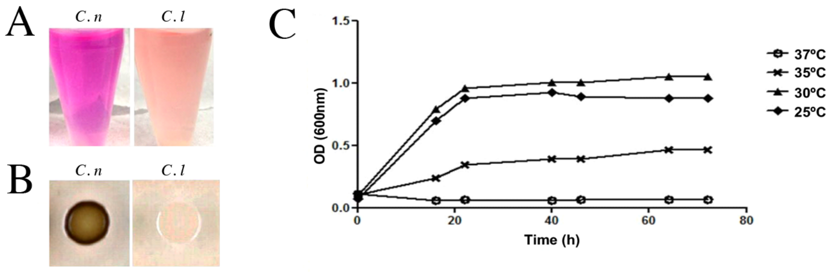

Figure S1: Physiological tests of *C. liquefaciens.* A) Urease test, showing a positive reaction for *C. neoformans*, and a negative reaction for *C.liquefaciens*. B) Melanization assay by incubation with L-dopa. Black colonies (indicative of melanization) were observed in *C. neoformans* cultures, but not in *C. liquefaciens* cultures, which were negative for melanization (only white colonies observed). C) Growth curves of *C. liquefaciens* at different temperatures, in minimal medium. X-axis correspond to time in hours. Y-axis correspond to optical density at 600 nm.
